# 

**Published:** 1924-09

**Authors:** 


### RECENT APPOINTMENTS.

### Medical Officers, &c.

Barker, Henry Lewis, M.D., etc.; Assistant Medical Officer of Health, Rotherham.

Bradley, Miss R., M.B., B.S., L.R.C.P., M.R.C.S.; Casualty Medical Officer, Hampstead General and North-West London Hospital, Haverstock Hill.

Campbell, Helen, M.B.; Assistant M.O., Warrington.

Cohen, Henry, M.B.; Assistant Honorary Physician, Royal Infirmary, Liverpool.

Dick, B. M., M.B., Ch.B.; House Surgeon, Hampstead General and North-West London Hospital, Haverstock Hill.

Edwards, Miss K. St. V., M.R.C.S., L.R.C.P.; Medical Officer, Surgical Department, Glanely Sanatorium, South Wales.

Enright, Francis, L.R.C.S., L.H., L.R.C.P., L.H., Resident M.O. Sussex County Hospital; Resident M.O. Brighton Borough Sanitorium.

Gray, A. O., F.R.C.S., L.R.C.P.; Gynæcologist, Hampstead General and North-West London Hospital, Haverstock Hill.

Hayward, Miss M. F., M.B., B.S.; Casualty Surgical Officer, Hampstead General and North-West London Hospital, Haverstock Hill.

Higson, W. D., M.B., Medical Officer, Liverpool Prison; Medical Officer, Maidstone Prison.

Hodgson, H. G., M.B., B.S., D.M.R.E.; Radiologist, Hampstead General and North-West London Hospital, Haverstock Hill.

Morgan, Leslie S., M.R.C.S., L.R.C.P., House Physician Hospital for Consumption and Diseases of Chest; Resident M.O., Hospital for Consumption and Diseases of the Chest.

Mercer, Leon Dalles, M.B., Ch.M., F.R.C.S.; Honorary Assistant Surgeon, Royal Hospital, Salford.

Murdock, Georgina, M.B., Ch.B., D.P.H., Assistant School M.O., Wigan; Assistant M.O., Wigan.

Reid, Alexander, M.B., Ch.B.; Resident M.O., Jersey General Hospital and Poor Law Infirmary.

Stobo, A., M.B., Ch.M.; House Physician, Hampstead General and North-West London Hospital, Haverstock Hill.

### Public Health and Hospital Appointments.

Anson, A. E.; Health Visitor and School Nurse, Middleton

Arnold, F.; Sanitary Inspector, Windsor.

Bennett, Ada M.; Superintendent, Q.V.J.I., East London.

Collard, Elsie, C.M.B.; Matron, Worthing Hospital.

Cooke, R. Gordon, M.B.; Senior Resident Medical Officer, Public Dispensary, Leeds.

Crofts, J. T.; Superintendent Sanitary Engineer, Bristol.

Croucher, Leonard; Sanitary Inspector, Dartford.

Culleton, Mary; Superintendent Health Visitor, West Suffolk.

Dawkins, Susan; Health Visitor and School Nurse, Exeter. £185.

Duncan, Isabel; Matron, Tuberculosis Pavilion, Scapa.

Eade, D. H., A.C.I.S., A.I.S.A., Acting Secretary, Ministry of Pensions Hospital, Shepherd's Bush; Secretary, All Saints' Hospital, Vauxhall Bridge Road.

Forbes, Thomas; Sanitary Inspector St. Marylebone. £190.

Goodwin, L. D., C.M.B.; Health Visitor, Isle of Ely.

Harland, H. J.; Sanitary Inspector, Margate.

Hinton, Alice L.; Health Visitor, Essex.

Logan, A.; Matron, Queen Victoria Hospital for Seamen, Las Palmas.

Marshall, E. M.; Health Visitor and School Nurse, Norwich.

McLennan, C. M., R.R.C.; Matron, Lochmaben Sanatorium and Infectious Diseases Hospital.

Morris, Edith M.; Superintendent, Q.V.J.I., Birmingham (Summer Hill Road).

Newton, Eleanor C. V., C.M.B.; Matron, Catherine Gladstone Maternity Home, Mancot Royal, Chester.

Prydderch, S.; Health Visitor and School Nurse, Denbighshire.

Shelswell, H. B., Assistant Secretary, General Hospital, Birmingham; Secretary, Jessop Hospital for Women, Sheffield.

Smith, Mary; Superintendent, Q.V.J.I., Fife.

Smith, Muriel; Health Visitor and School Nurse, Rhondda.

Stewart, Mary J.; Matron, Evan Fraser Hospital, Hull.

Tait, Agnes; Health Visitor, Berwickshire.

Townsend, Isabelle, Assistant Superintendent Stafford County Nursing Association; Health Visitor, Wiltshire

Underhill, Florence M.; Superintendent, Q.V.J.I., Huddersfield.

Wales, M.; Health Visitor, Isle of Ely.

Williams, F. H.; Sanitary Inspector, Liskeard.

Wood, H. St. J., Secretary, West Kent General Hospital, Maidstone; Secretary, General Hospital, Northampton.

---

### ANSWERS TO CORRESPONDENTS.

Overseas.—There is a hospital with 230 beds at Pretoria. Paying patients are received at 12s. 6d. per day. The institution is under Government control.

Vitamins.—Vitamin A. stimulates growth in children—lack of it may cause sore eyes; B. helps digestion and nerves; C. aids in the formation of bone and teeth. All three kinds are contained in cabbage, potato, carrots, tomatoes, oranges, liver, milk, and lean meat. A full list of vitamin values is given in this year's Report on the Present State of Knowledge of Accessory Food Factors (Vitamins), obtainable from H.M. Stationery Office for 4s. 6d.

The Lady Bird.—Your letter was received too late for publication in our last number. Moreover, you omitted to send your name and address.

Home Manager.—Yes, the University of London has a course of domestic science, so has the King's College for Women, Household and Social Science Department, Campden Hill Road, Kensington.

### Managerial Notices.

***All letters on Business matters must be addressed to the Manager, "The Hospital and Health Review," 28 and 29 Southampton Street, London, W.C. 2.***

**Rates for Subscription (payable in advance).**

| | | | |
|---|---|---|---|
| Three Months (including Postage) | | .. .. | 1s. 9d. |
| Six Months | do. | .. .. | 3s. 6d. |
| Twelve Months | do. | .. .. | 7s. 0d. |

**It is especially requested that remittances be made by Cheque or Postal Order and not Stamps.**

**Subscriptions, which may commence at any time, are payable in advance. Cheques and Post-Office Orders should be crossed Westminster Bank, Covent Garden Branch, and made payable to the Manager of The Hospital and Health Review.**

***Advertisements.*** **Scale of charges and particulars of special positions may be obtained on application to the Manager.**

***SUBSCRIPTION FORM.***

*Please send me* The Hospital and Health Review *for*.............. *months, commencing with your issue of* .................*, for which please find enclosed* ........

*Name* ..............................................

*Address* ..............................................

..............................................

*Date* ..............................................

*To*........ ..............................*Newsagent*

